# Chronic kidney disease linked to SARS-CoV-2 infection: a case report

**DOI:** 10.1186/s12882-021-02490-z

**Published:** 2021-08-10

**Authors:** Georges Tarris, Alexis de Rougemont, Marie-Anaïs Estienney, Julien Journet, Anne-Cécile Lariotte, Damien Aubignat, Jean-Michel Rebibou, Mathilde Funes De La Vega, Mathieu Legendre, Gael Belliot, Laurent Martin

**Affiliations:** 1 Department of Pathology, University Hospital of Dijon, F-21000 Dijon, France; 2 National Reference Centre for Gastroenteritis Viruses, Laboratory of Virology, University Hospital of Dijon, F-21000 Dijon, France; 3 Department of Nephrology, William Morey Hospital, F-71100 Chalon-sur-Saône, France; 4 Department of Nephrology, University Hospital of Dijon, F-21000 Dijon, France

**Keywords:** Coronavirus, COVID-19, SARS-CoV-2, Chronic viral replication, Chronic kidney disease, Immunocompromised, HBGA, Lewis antigens, Case report

## Abstract

**Background:**

The recent COVID-19 pandemic has raised concerns about patient diagnosis and follow-up of chronically ill patients. Patients suffering from chronic illnesses, concomitantly infected by SARS-CoV-2, globally tend to have a worse prognosis and poor outcomes. Renal tropism and acute kidney injury following SARS-CoV-2 infection has recently been described in the literature, with elevated mortality rates. Furthermore, patients with pre-existing chronic kidney disease, infected by SARS-CoV-2, should be monitored carefully. Here, we report the case of a 69-year-old patient with splenic marginal zone lymphoma, suffering from longstanding chronic kidney disease following SARS-CoV-2 infection.

**Case presentation:**

A 69-year-old male patient previously diagnosed with pulmonary embolism and splenic marginal zone lymphoma (Splenomegaly, Matutes 2/5, CD5 negative and CD23 positive), was admitted to the hospital with shortness of breath, fever and asthenia. A nasopharyngeal swab test was performed in addition to a CT-scan, which confirmed SARS-CoV-2 infection. Blood creatinine increased following SARS-CoV-2 infection at 130 μmol/l, with usual values at 95 μmol/l. The patient was discharged at home with rest and symptomatic medical treatment (paracetamol and hydration), then readmitted to the hospital in August 2020. A kidney biopsy was therefore conducted as blood creatinine levels were abnormally elevated. Immunodetection performed in a renal biopsy specimen confirmed co-localization of SARS-CoV2 nucleocapsid and protease 3C proteins with ACE2, Lewis x and sialyl-Lewis x antigens in proximal convoluted tubules and podocytes. Co-localization of structural and non-structural viral proteins clearly demonstrated viral replication in proximal convoluted tubules in this chronically ill patient. Additionally, we observed the co-localization of sialyl-Lewis x and ACE2 receptors in the same proximal convoluted tubules. Reverse Transcriptase-Polymerase Chain Reaction test performed on the kidney biopsy was negative, with very low Ct levels (above 40). The patient was finally readmitted to the haematology department for initiation of chemotherapy, including CHOP protocol and Rituximab.

**Conclusions:**

Our case emphasizes on the importance of monitoring kidney function in immunosuppressed patients and patients suffering from cancer following SARS-CoV-2 infection, through histological screening. Further studies will be required to decipher the mechanisms underlying chronic kidney disease and the putative role of sialyl-Lewis x and HBGA during SARS-CoV-2 infection.

**Supplementary Information:**

The online version contains supplementary material available at 10.1186/s12882-021-02490-z.

## Background

Since December 2019, the COVID-19 pandemic has become a major public health issue worldwide and the source of substantial healthcare costs [1]. Severe Acute Respiratory Syndrome related Coronavirus-2 (SARS-CoV-2) belongs to the Sarbecovirus subgenus along with Severe Acute Respiratory Syndrome related Coronavirus-1 (SARS-CoV-1) [2]. SARS-CoV-2 possesses a large spectrum of virulence, varying from asymptomatic infection to severe acute respiratory syndrome (SARS) and multi-organ failure linked to cytokine storm, with possible Kawasaki-like syndromes and auto-immune manifestations, linked to interleukin 6 (IL-6) hypersecretion [3, 4]. Autopsy case series have shown several types of lesions linked to SARS-CoV-2 infection, especially alveolar damage with viral replication in pneumocytes, but also viral replication in the kidneys with podocyte and proximal tubular involvement [5, 6]. Acute kidney injury has also been associated with severe COVID-19 infection and increased in-hospital mortality [7]. SARS-CoV-2 mainly uses the angiotensin-converting enzyme (ACE2) as an entry point into infected cells prior to protein priming by transmembrane protease serine 2 (TMPRSS2) [8]. Blood group antigen polymorphisms might also modulate virus binding and infectivity, as individuals with blood group A and B are potentially more susceptible to SARS-CoV-2 infections [9, 10]. Epidemiological studies recently illustrated the association between SARS-CoV-2 severe renal dysfunction and the occurrence of chronic comorbidities [11]. Here, we demonstrate the occurrence of SARS-CoV-2 chronic replication in the kidney of a comorbid patient, leading to longstanding chronic kidney disease (CKD).

## Case presentation

In April 2020, a 69-year-old man with blood group A was admitted to the William-Morey General Hospital (Chalon-sur-Saône, France), with shortness of breath, fever and fatigue. His personal medical history included an indolent splenic marginal zone lymphoma (SMZL) of Matutes score 2 (splenomegaly, CD5 negativity and CD23 positivity), diagnosed following an idiopathic pulmonary embolism in December 2019, with no previous history of lung disease [12]. The patient was initially treated with apixaban for his pulmonary embolism. At the emergency room, a thoracic CT-scan showed bilateral ground-glass pulmonary infiltrates suggesting SARS-CoV-2 infection which was later confirmed by a nasopharyngeal swab RT-qPCR test. Blood tests showed elevated blood C-reactive protein (25 mg/ml) and ferritin (514 ng/dl) levels, associated with a deep lymphopenia (0,03 G/L), but normal electrolytes, urea and creatinine levels. The patient was carefully monitored and his condition rapidly improved with appropriate hydration and ventilation. One week later, the patient was discharged at home with routine guidelines following SARS-CoV-2 infection, including rest at home, hand hygiene, avoidance of contact with the surroundings, paracetamol intake and appropriate hydration in case of symptoms. During the following weeks, the patient suffered from dyspnea at exertion and chronic invalidating fatigue at home, later confirmed by his family physician. Serologies for SARS-CoV-2 performed in June 2020 were negative.

In August 2020, the patient was again admitted to the hospital due to intense exhaustion and significant weight loss (6 kg) during the 2 weeks prior to his admission, associated with mild fever, breathlessness and daytime sweating. Laboratory tests showed a microcytic anaemia (10.5 g/dl), a mild hypogammaglobulinemia associated with a persistent lymphopenia (0.6 G/L) and an altered renal function with elevated serum creatinine (167 μmol/l) and urea levels (13.7 mmol/l). The estimated glomerular filtration rate (eGFR) was 35 ml/min/1.73m^2^ using the CKD-EPI formula. Urine analysis revealed mild proteinuria at 0.09 g/24 h.

**Fig. 1:**
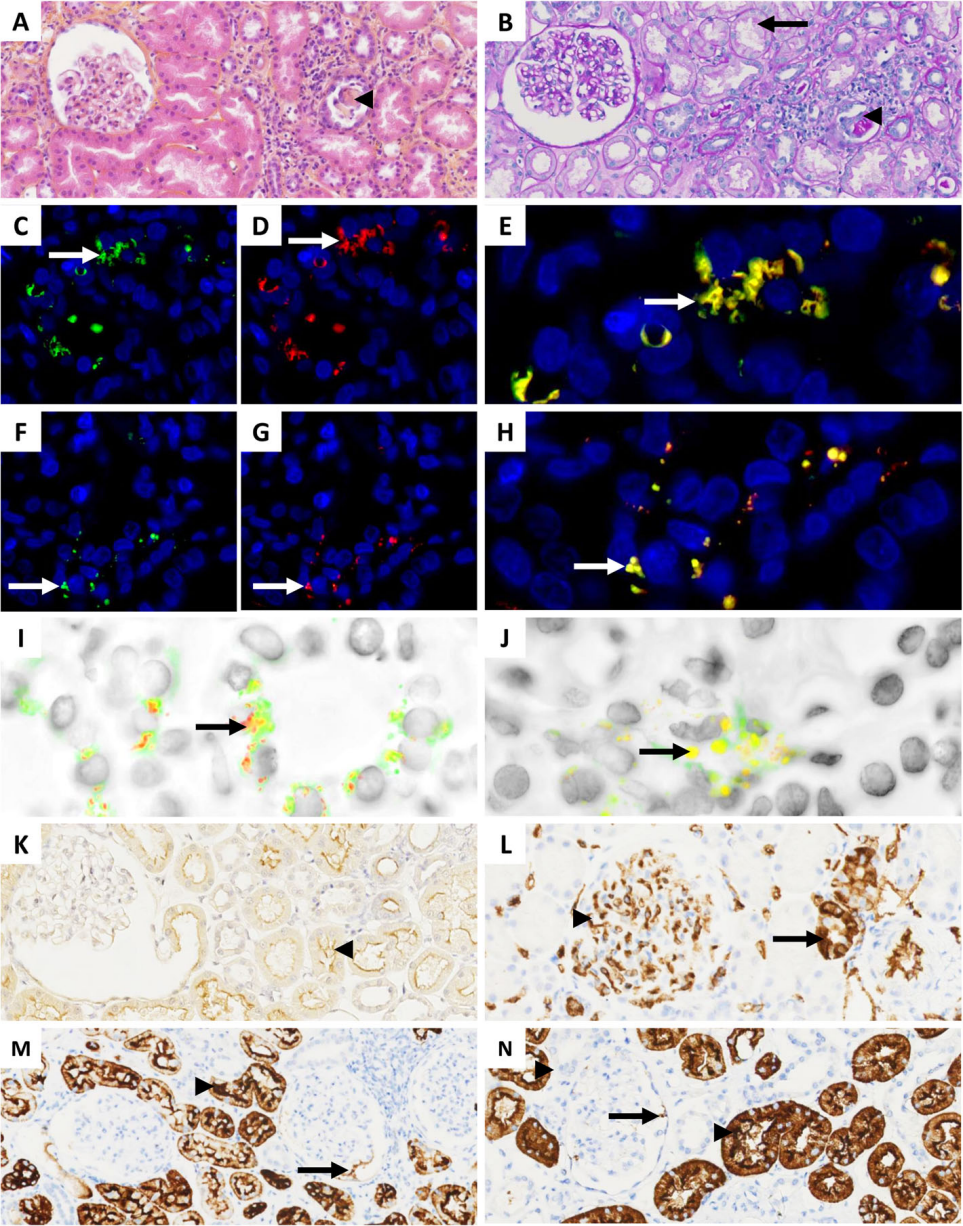
Histopathology and immunodetection assays in formalin-fixed paraffin embedded kidney biopsy from a 69-year-old patient suffering from chronic COVID-19 infection. **A** (HES, × 200) and **B** (PAS, × 200): grade I interstitial fibrosis associated with grade I tubular atrophy without glomerular damage; focal desquamation of the brush border of epithelial cells of proximal convoluted tubules (arrow), associated with focal sloughing of epithelial cells of proximal convoluted tubules with intra-tubular cell casts (arrowheads); **C, D, E** and **I** (× 600): co-detection of SARS-CoV-2 nucleoprotein (green fluorescence) and protease-3C (red fluorescence) in epithelial cells of proximal convoluted tubules (arrows) using anti-NP and anti-Prot3C mAb; **F, G, H** and **J** (× 600): co-detection of SARS-CoV-2 nucleoprotein (green fluorescence) and protease3C (red fluorescence) in the glomerular apparatus (arrows) using anti-NP and anti-Prot3C mAb; **K** (× 200): ACE2 detection on the brush border of epithelial cells of proximal convoluted tubules (arrowhead) using anti-ACE2 mAb. Positive detection is shown by brown staining; **L** (× 200): A antigen expression in glomerular capillary walls (arrowhead) and distal convoluted tubules (arrow) using anti-A mAb. Positive detection is shown by brown staining; **M** (× 200): Le^x^ antigen expression in proximal convoluted tubules (arrowhead) and podocytes (arrow) using anti-Le^x^ mAb. Positive detection is shown by brown staining; **N** (× 200): sialyl-Le^x^ antigen expression in proximal convoluted tubules (arrowhead) and podocytes (arrow). Positive detection is shown by brown staining

A kidney biopsy was then performed to explore the chronic kidney disease and sent to the Pathology Department of the University Hospital of Dijon (Bourgogne, France) for further investigation. Analysis of the kidney biopsy was conducted according to previously established criteria [13]. Histopathology showed 9 normal glomeruli and 2 obsolescent glomeruli with cortical grade I fibrosis and grade I tubular atrophy with focal interstitial inflammation, focal sloughing of epithelial cells of proximal convoluted tubules and intra-tubular cell casts, which indicated reversible and focal tubular necrosis (Fig. 1A and B). Periodic Acid Schiff (PAS) staining revealed desquamation of the brush border of proximal convoluted tubules (Fig. 1B). Silver impregnation (Marinozzi) showed otherwise normal glomerular basement membranes. Congo red staining and direct immunofluorescence for IgA, IgM, IgG, albumin, fibrinogen, C3, C4c, C1q, kappa and lambda light chains were all negative.

The presence of focal tubular necrosis was initially attributed to possible dehydration in a patient which manifested daytime sweating, as clinical examination and history taking ruled out other causes of tubular injury. In the context of tubular lesions in a patient suffering from symptom exacerbation possibly related to SARS-CoV-2 infection, the possibility of direct tubular damage due to SARS-CoV-2 replication was explored through SARS-CoV-2 immunohistological analysis on biopsy slides following described protocols (Supplemental data). Double fluorescent staining revealed cytoplasmic co-localization of SARS-CoV-2 nucleoprotein and protease-3C in proximal convoluted tubules and podocytes, demonstrating virus binding and replication (Figs. 1C -J). Immunohistochemistry demonstrated the expression of ACE2 in the cytoplasm of epithelial cells bordering the proximal convoluted tubules (Fig. 1K), A antigen in capillary walls and distal convoluted tubules (Fig. 1L), and Le^x^ and sialyl-Le^x^ (CD15s) in the proximal convoluted tubules and podocytes (Fig. 1M and N).

The patient received effective and complete anticoagulative therapy by apixaban from December 2019 to April 2020. The patient stayed at the hospital for one week, with intravenous fluids and careful monitoring until his renal function improved enough to be sent home with appropriate symptomatic care. At present, the patient continues to complain of persistent dyspnoea on exertion and chronic fatigue, possibly caused by aggravation of SMZL or persistent SARS-CoV-2 infection. The patient was later readmitted to the haematology department for initiation of chemotherapy including Cyclophosphamide, Hydroxydaunorubicin, Oncovin and Prednisone (CHOP), without administration of Rituximab due to chronic kidney disease partially induced by the previous SARS-CoV-2 infection. Testing of SARS-CoV-2 using serologies and nasopharyngeal swabs were negative in January 2021.

**Fig. 2:**
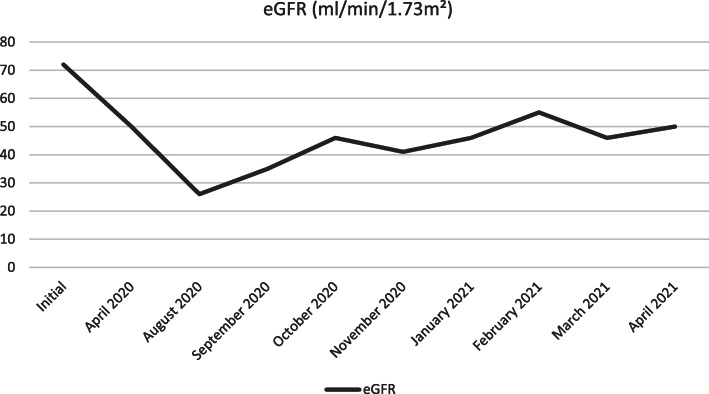
Trends of renal function in a patient suffering from Chronic Kidney Disease following SARS-CoV-2 infection. eGFR: estimated Glomerular Filtration Rate

Before the initial episode of infection, blood creatinine levels were estimated at 95 μmol/l (eGFR was initially measured at 72 ml/min/1.73m^2^). Blood creatinine levels increased to 130 μmol/l in April 2020 (eGFR: 50 ml/min/1.73m^2^) up to 230 μmol/l in August 2020 (eGFR: 26 ml/min/1.73m^2^). Thereafter, the renal function was sustainably altered, with blood creatinine values ranging from 120 to 180 μmol/l (eGFR from 35 to 55 ml/min/1.73m^2^) (Fig. 2). Of note, no measurements of renal function were conducted between April 2020 and August 2020.

## Discussion and conclusions

Based on autopsy tissue samples, the ability of SARS-CoV2 to bind, replicate and induce cellular damage in podocytes and proximal convoluted tubules, where ACE2 is highly expressed, have been suggested [5, 6]. Recent data has described the mechanisms underlying SARS-CoV-2 replication and kidney damage involving innate immunity and coagulation pathways [14, 15]. Indeed, SARS-CoV-2 binding to ACE2 induces CD4+ and CD8+ lymphocyte depletion as seen in our case, in addition to the SMZL comorbidity in this patient, that might have aggravated lymphocyte depletion [16]. Moreover, elevated ferritin levels and anaemia could reflect chronic inflammation linked to SARS-CoV-2 infection, and could be used as a marker of persistent infection [17, 18]. The expression of histo-blood group antigens (HBGA) in nephrons could also contribute to SARS-CoV-2 binding, as Le^x^ and CD15s are highly expressed in proximal convoluted tubules [19]. Indeed, in vitro studies have shown the high affinity of SARS-CoV-2 spike glycoprotein towards CD15s and other sialylated molecules with differential ligand affinity, as described with MERS-CoV and SARS-CoV-1 [20]. Additionally, the role of the blood group A antigen in SARS-CoV-2 susceptibility was not highlighted in our case, as its identification in capillary walls and distal convoluted tubules was not related to SARS-CoV-2 binding and replication [10, 19, 21].

Further studies will be required in order to understand the mechanisms underlying chronic kidney disease associated with SARS-CoV-2 binding and replication in kidney cells. Kemp et al. recently described the possibility of SARS-CoV-2 to chronically infect a 70-year-old patient suffering from B-cell lymphoma, and to replicate in organs with possible de novo mutations of the spike protein at different sites of replication [22]. Regarding the occurrence of renal damage linked to SARS-CoV-2 infections, the high incidence of acute kidney injury (AKI) and acute tubular necrosis with SARS-CoV-2 replication in proximal convoluted tubules in severe and lethal forms of SARS-CoV-2 infections has previously been documented in the literature [23]. Actual scientific data points towards CKD as an important risk factor of severe and prolonged SARS-CoV-2 infection [11, 24, 25]. To our knowledge, data concerning the mechanisms underlying the occurrence of CKD following SARS-CoV-2 infection remain scarce. Current guidelines raise concern about appropriate surveillance of kidney function in patients who suffered from SARS-CoV-2 infection, indicating to refer patients with symptoms of exacerbation of chronic kidney disease or heavy proteinuria, and regular blood and urine analyses [26, 27]. The importance of histological screening in patients with CKD following SARS-CoV-2 infection remain an important point to discuss in comorbid patients recovering from SARS-CoV-2 infection. Until vaccination becomes fully developed worldwide, more complete data will be needed in order to provide appropriate care and treatment for comorbid patients suffering from chronic kidney disease following SARS-CoV-2 infection.

## Supplementary Information

**Additional file 1.**

## Data Availability

The images and digitized histology analysed during the current study are available from the corresponding author on reasonable request.

## Abbreviations

ACE2: Angiotensin-Converting Enzyme 2
AKI: Acute Kidney Injury
C1q: Complement component 1q
C3: Complement component 3
C4c: Complement component 4
Ct: Cycle threshold
CT-scan: Computed-Tomography scan
CD15s: Sialyl-lewis x
CD4: Cluster of Differentiation 4
CD5: Cluster of Differentiation 5
CD23: Cluster of Differentiation 23
CD8: Cluster of Differentiation 8
CKD: Chronic Kidney Disease
CKD-EPI: Chronic Kidney Diseases – Epidemiology Collaboration
COVID-19: Coronavirus Disease of 2019
eGFR: Estimated Glomerular Filtration Rate
HBGA: Histo-Blood Group Antigens
IgA: Immunoglobulin A
IgG: Immunoglobulin G
IgM: Immunoglobulin M
IL-6: Interleukin 6
IL-8: Interleukin 8
Le^x^: Lewis x
mAb: Monoclonal antibody
MERS-CoV: Middle East Respiratory Syndrome - Coronavirus
PAS: Periodic Acid Schiff
SARS-CoV-1: Severe Acute Respiratory Syndrome – Coronavirus 1
SARS-CoV-2: Severe Acute Respiratory Syndrome – Coronavirus 2
SMZL: Splenic Marginal Zone Lymphoma
TMPRSS2: Transmembrane Protease Serine 2

## References

[1] Zhu H, Wei L, Niu P. The novel coronavirus outbreak in Wuhan, China. Glob Health Res Policy. 2020;5:6. 10.1186/s41256-020-00135-6. 10.1186/s41256-020-00135-6 PMC7050114 32226823

[2] Coronaviridae Study Group of the International Committee on Taxonomy of Viruses (2020). The species severe acute respiratory syndrome-related coronavirus: classifying 2019-nCoV and naming it SARS-CoV-2. Nat Microbiol.

[3] Tang Y, Liu J, Zhang D, Xu Z, Ji J, Wen C (2020). Cytokine storm in COVID-19: the current evidence and treatment strategies. Front Immunol.

[4] Long Q-X, Tang X-J, Shi Q-L, Li Q, Deng H-J, Yuan J, Hu JL, Xu W, Zhang Y, Lv FJ, Su K, Zhang F, Gong J, Wu B, Liu XM, Li JJ, Qiu JF, Chen J, Huang AL (2020). Clinical and immunological assessment of asymptomatic SARS-CoV-2 infections. Nat Med.

[5] Puelles VG, Lütgehetmann M, Lindenmeyer MT, Sperhake JP, Wong MN, Allweiss L, Chilla S, Heinemann A, Wanner N, Liu S, Braun F, Lu S, Pfefferle S, Schröder AS, Edler C, Gross O, Glatzel M, Wichmann D, Wiech T, Kluge S, Pueschel K, Aepfelbacher M, Huber TB (2020). Multiorgan and renal tropism of SARS-CoV-2. N Engl J Med.

[6] Su H, Yang M, Wan C, Yi L-X, Tang F, Zhu H-Y, Yi F, Yang HC, Fogo AB, Nie X, Zhang C (2020). Renal histopathological analysis of 26 postmortem findings of patients with COVID-19 in China. Kidney Int.

[7] Cheng Y, Luo R, Wang K, Zhang M, Wang Z, Dong L, Li J, Yao Y, Ge S, Xu G (2020). Kidney disease is associated with in-hospital death of patients with COVID-19. Kidney Int.

[8] Shang J, Wan Y, Luo C, Ye G, Geng Q, Auerbach A, Li F (2020). Cell entry mechanisms of SARS-CoV-2. Proc Natl Acad Sci U S A.

[9] Yamamoto F, Yamamoto M, Muñiz-Diaz E (2021). Blood group ABO polymorphism inhibits SARS-CoV-2 infection and affects COVID-19 progression. Vox Sang.

[10] Breiman A, Ruvën-Clouet N, Le Pendu J (2020). Harnessing the natural anti-glycan immune response to limit the transmission of enveloped viruses such as SARS-CoV-2. PLoS Pathog.

[11] Guan W-J, Liang W-H, Zhao Y, Liang H-R, Chen Z-S, Li Y-M, Liu XQ, Chen RC, Tang CL, Wang T, Ou CQ, Li L, Chen PY, Sang L, Wang W, Li JF, Li CC, Ou LM, Cheng B, Xiong S, Ni ZY, Xiang J, Hu Y, Liu L, Shan H, Lei CL, Peng YX, Wei L, Liu Y, Hu YH, Peng P, Wang JM, Liu JY, Chen Z, Li G, Zheng ZJ, Qiu SQ, Luo J, Ye CJ, Zhu SY, Cheng LL, Ye F, Li SY, Zheng JP, Zhang NF, Zhong NS, He JX, China Medical Treatment Expert Group for COVID-19 (2020). Comorbidity and its impact on 1590 patients with COVID-19 in China: a nationwide analysis. Eur Respir J.

[12] Matutes E (2013). Splenic marginal zone lymphoma: disease features and management. Expert Rev Hematol.

[13] Walker PD, Cavallo T, Bonsib SM, Ad Hoc Committee on Renal Biopsy Guidelines of the Renal Pathology Society (2004). Practice guidelines for the renal biopsy. Mod Pathol.

[14] Chen G, Wu D, Guo W, Cao Y, Huang D, Wang H, Wang T, Zhang X, Chen H, Yu H, Zhang X, Zhang M, Wu S, Song J, Chen T, Han M, Li S, Luo X, Zhao J, Ning Q (2020). Clinical and immunological features of severe and moderate coronavirus disease 2019. J Clin Invest.

[15] Giannis D, Ziogas IA, Gianni P (2020). Coagulation disorders in coronavirus infected patients: COVID-19, SARS-CoV-1, MERS-CoV and lessons from the past. J Clin Virol.

[16] Arcaini L, Rossi D, Paulli M (2016). Splenic marginal zone lymphoma: from genetics to management. Blood..

[17] Kappert K, Jahić A, Tauber R (2020). Assessment of serum ferritin as a biomarker in COVID-19: bystander or participant? Insights by comparison with other infectious and non-infectious diseases. Biomarkers.

[18] Perricone C, Bartoloni E, Bursi R, Cafaro G, Guidelli GM, Shoenfeld Y, Gerli R (2020). COVID-19 as part of the hyperferritinemic syndromes: the role of iron depletion therapy. Immunol Res.

[19] Ravn V, Dabelsteen E (2000). Tissue distribution of histo-blood group antigens. APMIS Acta Pathol Microbiol Immunol Scand.

[20] Awasthi M, Gulati S, Sarkar DP, Tiwari S, Kateriya S, Ranjan P, et al. The Sialoside-binding pocket of SARS-CoV-2 spike glycoprotein structurally resembles MERS-CoV. Viruses. 2020;12(9). 10.3390/v12090909. 10.3390/v12090909 PMC7551769 32825063

[21] Golinelli D, Boetto E, Maietti E, Fantini MP (2020). The association between ABO blood group and SARS-CoV-2 infection: a meta-analysis. PLoS One.

[22] Kemp SA, Collier DA, Datir RP, Ferreira IATM, Gayed S, Jahun A (2021). SARS-CoV-2 evolution during treatment of chronic infection. Nature..

[23] Diao B, Wang C, Wang R, Feng Z, Zhang J, Yang H, Tan Y, Wang H, Wang C, Liu L, Liu Y, Liu Y, Wang G, Yuan Z, Hou X, Ren L, Wu Y, Chen Y (2021). Human kidney is a target for novel severe acute respiratory syndrome coronavirus 2 infection. Nat Commun.

[24] O’Sullivan ED, Lees JS, Howie KL, Pugh D, Gillis KA, Traynor JP (2021). Prolonged SARS-CoV-2 viral shedding in patients with chronic kidney disease. Nephrol Carlton Vic.

[25] ERA-EDTA Council (2021). ERACODA working group. Chronic kidney disease is a key risk factor for severe COVID-19: a call to action by the ERA-EDTA. Nephrol Dial Transplant.

[26] COVID-19 rapid guideline: chronic kidney disease. London: National Institute for Health and Care Excellence (UK); 2020 May 15. (NICE Guideline, No. 176.) 33400454

[27] Kant S, Menez SP, Hanouneh M, Fine DM, Crews DC, Brennan DC, Sperati CJ, Jaar BG (2020). The COVID-19 nephrology compendium: AKI, CKD, ESKD and transplantation. BMC Nephrol.

