# Supplementary material for: Chronic kidney disease linked to SARS-CoV-2 infection: a case report
Source: BMC Nephrol. 2021 Aug 10;22:278. doi: 10.1186/s12882-021-02490-z (PMC8353426; doi:10.1186/s12882-021-02490-z)
Supplement: Supplementary file 1 — Additional file 1. [file 12882_2021_2490_MOESM1_ESM.docx]

**SUPPLEMENTAL DATA**

**Material and methods**

COVID-19 detection was performed on formalin-fixed paraffin-embedded 4 µm-thick slides. Slides were immersed in 10 mM sodium citrate, 0.05% Tween 20, pH 6.0 heated at 97°C for 10 minutes, then covered with 0.2% Triton X-100 diluted in PBS for 10 minutes. Sections were finally rinsed and covered with PBS/BSA 1% for 2 hours at room temperature (RT) prior to antibody incubation. Primary antibodies were mouse monoclonal antibodies directed against the SARS-CoV-2 Nucleoprotein (clone 40143-MM08, Sinobiological, China) diluted at 1/500, and rabbit polyclonal antibodies directed against the SARS-CoV Protease 3C (Reference NBP1-78110, Novus Biologicals, USA) showing 100% sequence homology with the SARS-CoV-2 Protease. Anti-ACE2 (clone T24, Sinobiological, China) and anti-sialyl-Lewis x (clone C-SLEX, Becton Dickinson, USA) antibodies were diluted at 1/5000 and 1/500, respectively. Double fluorescent staining was performed with Alexa488-conjugated anti-mouse antibodies and Alexa568-conjugated anti-rabbit antibodies, diluted at 1/2000 and incubated for 45 minutes at room temperature. Slides were mounted with ProLong Gold Antifade reagent (Invitrogen, USA) containing 4’6’-diamino-2-phenylindole (DAPI). Lewis x (Le^x^) and A antigens were detected with mouse anti-CD15 (clone Carb-3, Dako, USA) and anti-A antibodies (clone 9113D10, Diagast, France) diluted at 1/50 and 1/1000, respectively. Antibody detection was performed on an Omnis automaton (Dako, USA) according to manufacturer recommendations.
